# Modeling and Simulation of a Multizone Circulating Reactor for Polyethylene Production with Internal Cooling

**DOI:** 10.3390/polym15183741

**Published:** 2023-09-13

**Authors:** Nayef Ghasem

**Affiliations:** Department of Chemical and Petroleum Engineering, United Arab Emirates University, Al-Ain P.O. Box 15551, United Arab Emirates; nayef@uaeu.ac.ae

**Keywords:** multizone circulating reactor, modeling, simulation, polymerization, polyethylene, CFD

## Abstract

Polyolefins play a role in industries and are typically manufactured using two types of reactors: high-pressure tubular reactors and fluidized bed reactors. An innovative technology called the Multizone Circulating reactor (MZCR) has emerged, which introduces an innovative approach with interconnected polymerization zones creating a continuous loop of polymer flow. This study focuses on modeling and simulating ethylene gas phase polymerization within the MZCR in the presence of internal cooling to gain insights into its behavior. To achieve this, a comprehensive computational fluid dynamics (CFD) simulation was developed. It considered momentum, material, and energy balance aspects. The model equations were solved using the finite difference method in COMSOL Multiphysics version 6.1. The investigation primarily focused on studying the impact of incorporating a cooler into the riser section on the temperature profile within the reactor and ethylene conversion. The presence of this cooler resulted in a reduction in temperature change along the riser from approximately 8.0 °C to 4.0 °C. Moreover, it led to an increase of 7%, in ethylene single-pass conversion.

## 1. Introduction

**Figure 1:**
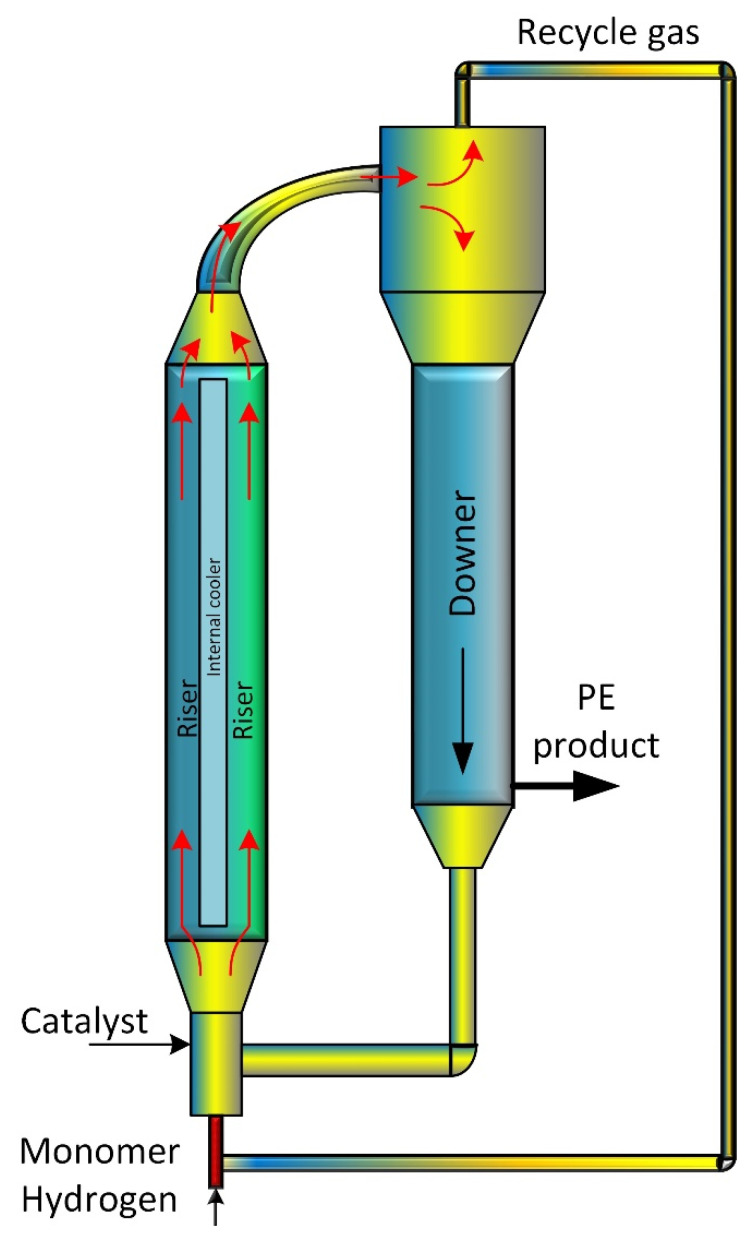
Schematic diagram of MZCR with internal cooler used in polyethylene production. The external arrows represent the feed, catalyst feed streams, and polyethylene (PE) product streams, while the internal arrows illustrate the flow direction of both gas and polymer particles.

Polyolefins are polymers with widespread application through varied industries. Polyethylene and polypropylene are kinds of polyolefins derived through the polymerization process of ethylene and propylene gas, respectively. Polyethylene polymer is a remarkable material highly regarded for its remarkable resistance to chemicals, low moisture absorption, and easy to process [1]. The MZCR is a cutting-edge technology used in the manufacturing of polyethylene utilized polymer in various industries (Figure 1). This reactor design incorporates zones with temperature and pressure conditions allowing for precise control over the process of polymerization. One of the advantages of this reactor is its internal cooling mechanism, which effectively dissipates the heat generated during the reactions involved in polymerization. This cooling mechanism guarantees a uniform temperature distribution within the reactor preventing any areas from becoming excessively hot and ensuring a controlled production process. By utilizing the Multizone Circulating Reactor manufacturers can achieve higher rates of polyethylene production while maintaining product quality. Additionally, the improved cooling capabilities contribute to reduced energy consumption, making this process more environmentally friendly and cost-effective [2]. Polyethylene polymers possess flexibility and versatility which makes them highly suitable for an array of uses across different industries. Particularly, they prove to be a substitute for polypropylene when it comes to packaging, tube fabrication, and the creation of containers [3]. Particularly, polyethylene is acknowledged for its outstanding chemical resistance, insignificant moisture absorption, and easy processability. Polypropylene proved highly for fabricated polymeric membranes used in carbon dioxide absorption [4,5]. Polyethylene stands as an adaptable and widely employed plastic [6]. Additionally, the use of Multizone Rotary Reactor technology (Figure 1) improves polymer properties with the help of the constant circulation of developing polymer granules between two fluidization systems [7,8,9].

In contrast, to a conventional fluidized bed reactor, MZCR has the capability to produce polymers of different molecular weights within an individual particle [10,11]. By circling the polymer granules, MZCR maintains the fine mixing of different polymers between broad and incomplete molecular weight distributions and the blending of incompatible polymers [12]. Modeling the polymerization of the ethylene gas phase in the MZCR showed good agreement with the other models [9]. The study investigated the influences of the recycling ratio and the effect of the operating parameters, displaying the important inspiration of the hydrogen concentration at the bottom on the polymer properties [2]. A comprehensive assessment was conducted of the newest improvements in gas-phase propylene polymerization reactors, with definite attention to multizone reactors. It investigates the evolution made in multiscale modeling incorporating kinetics, transport, and thermodynamics. Furthermore, it stresses the tasks encountered and recognizes vague matters in the domain of mathematical multiscale modeling [13]. Ge et al. [14] investigate the effect of electrostatic consequences on the hydrodynamics within the riser of circulating fluidized bed (CFB) reactors used for polypropylene production. Experimental and simulation outcomes show that electrostatics increase solids holdup in the riser by more than 20% in fast fluidization. The mechanism behind these influences is attributed to the increased velocity of the radial particle and particle accumulation near the wall, particularly in the dense phase region [15]. A two-fluid model relating to the Eulerian-Eulerian polypropylene propagation and population balance model (PBM) is developed to describe gas-solid two-phase flow in an MZCR, combining polymerization kinetics. The model predicts the complete field in the MZCR considering solid phase polydispersity, analyzes temperature distribution considering polymerization reaction, and distinguishes flow behaviors between a circulating fluidized bed reactor (CFBR) and MZCR, highlighting significant differences in temperature distribution between the two modes [16]. Yan et al. [17] extended the CFD modeling of the propylene polymerization process in an MZCR to design a gas-barrier system. A Eulerian-Eulerian model integrating the kinetic theory of granular flow describes the gas-solid flow and optimizes the gas-barrier inlet configuration, following in a suitable four-way tangential gas-barrier system for the MZCR [18,19]. The study reveals the propylene polymerization of an established approach in chemical engineering to a new field, opening new possibilities in olefin polymerization research. Modeling propylene gas-phase polymerization in MZCR confirmed the accuracy of the developed model, which was compared with models available in the literature. The study explored the effect of the recycling ratio on monomer concentration and polypropylene’s relative molecular mass, along with conducting a sensitivity analysis of propylene and hydrogen concentrations [20]. Results showed that hydrogen concentration in the downer significantly impacted the polymer’s average relative molecular mass [21]. A polyethylene circulating fluidized reactor was modeled by combining hydrodynamics for the riser and downer with a kinetic model based on moment equations [22]. The model successfully predicted various parameters’ profiles, including cluster velocity, bed porosity, active site concentration, gas-phase components, molecular weights, and reactor temperature. The study demonstrated that controlling hydrodynamic conditions, such as gas velocity and solid circulation rate, allows for manipulating monomer consumption and molecular weight, influencing reactor behavior and production properties. A validated Eulerian-Eulerian model simulates gas-solid two-phase flows in a multizone circulating propylene polymerization reactor. The model captures the distinct flow behaviors in the riser and downer sections, showing the significant impact of riser outlet configuration and gas velocity. This contributes to optimizing the reactor’s design and operation [23]. Spherizgone process technology develops polypropylene production via an MZCR, improving polymer homogeneity and assisting segregated products with outstanding properties. Polypropylene’s versatility and sustainability make it a practical replacement for conventional materials and standard polymers [24]. In the conducted search, a dynamic model was utilized to completely analyze the dynamics involved in MZCR during the production process of polyethylene. The focus was particularly directed toward studying the copolymerization of ethylene with butene. Through rigorous parameter sensitivity analyses, the exploration shed light on the reactor’s susceptibility to variations in cooling water temperature. Notably, it emphasized the criticality of temperature control to mitigate undesired temperature fluctuations and potential complications arising from polymer melting issues [25]. A dynamic model is developed to analyze the reactor’s behavior, concentrating on the copolymerization of ethylene with butene. Parameter sensitivity analyses explain computer-simulated time responses for reactor temperature, molecular weight, catalyst feed rate, and monomer concentration along the reactor length [26]. A mathematical model is established to simulate the gas-phase polymerization process in the reactor, contemplating different gas-phase compositions in connected polymerization zones. Simulations reveal that an extensive range of product characteristics can be accomplished by adapting reactor operation conditions, with highly active catalysts being necessary for increasing the reactor’s benefits. This review targets the impact-modified polypropylene technologies, emphasizing the recently developed MZCR by Basel [27]. A comparison is made between the influence of polypropylene produced with MZCR, and multiphase polypropylene copolymers made using traditional methods. The review recommends further studies on in-situ reactor synthesis of impact polypropylene [28]. A mathematical model for the multilane circulating reactor was introduced, demonstrating its operational characteristics, and managing a parameter sensitivity analysis. The simulations were conducted with an emphasis on polyethylene production. A mathematical model for the reactor and simulations exploring the introduction of a gas barrier in the downer section and its implications on polymer characteristics. Various CFD models were developed to describe the concentration and the hydrodynamics in the MZCR [16,17,23,29]. In all these models, the cooling was performed using an external heat exchange. In the present work. A special device called an internal simple heat exchanger is added at the beginning of the polymerization reactor’s riser. The addition of the cooler helps control and improve the temperature right from the start of the reaction. With this simple device, it can better manage the process, distribute heat evenly, and make the reactor work more efficiently. The research findings offer exciting possibilities for advancing the polymerization process in industry.

## 2. Model Development

In the development of the MZCR model, fundamental principles of material balance, momentum balance, and heat transfer were employed. The fluid flow patterns of the gas and solids in both the riser and the downer of the MZCR are assumed to be plug flows considering radial gradients and axial dispersion. 

### 2.1. Material Balance

The material balance equation describes the conservation of mass within the reactor, accounting for the inflow and outflow of monomers, catalysts, solvents, and other components.

The overall molar monomer balances in cylindrical coordinates, neglecting concentration change in the angular direction [30].

(1) $$\frac{\partial C_{i}}{\partial t}=\nabla \cdot D_{i}\nabla c_{i}-u\cdot \nabla c_{i}+R_{i}$$

The general material balance equation can be represented in a more concise and comprehensive form.

(2) $$\frac{\partial C_{i}}{\partial t}=\frac{\partial }{\partial x}\left(D_{i}\frac{\partial C_{i}}{\partial x}\right)+\frac{\partial }{\partial y}\left(D_{i}\frac{\partial C_{i}}{\partial y}\right)+\frac{\partial }{\partial z}\left(D_{i}\frac{\partial C_{i}}{\partial z}\right)-u_{x}\frac{\partial C_{i}}{\partial x}-u_{y}\frac{\partial C_{i}}{\partial y}-u_{z}\frac{\partial C_{i}}{\partial z}+R_{i}$$

Initial conditions

$$C_{i}=C_{io}\;\mathrm{at}\;t=0$$

Boundary conditions:

$\frac{\partial C_{i}}{\partial x}=\frac{\partial C_{i}}{\partial x}=0$ at the walls of the reactor.

In these equations, $C_{i}$ represents the concentration of the dilute species, ∇ is the del operator, representing the gradient in space (∇ = (∂/∂x, ∂/∂y, ∂/∂z)), $D_{i}$ is the diffusion coefficient of the species, u is the flow velocity vector. $R_{i}$ represent the rate of generation or consumption of species “i” due to chemical reactions or other processes. The ethylene consumption ($R_{e}$) considering a single type of catalyst and well-mixed reactor [11,31].

(3) $$R_{e}=-k_{p}C_{e}y(1-\varepsilon )$$

where $k_{p}=k_{po}\exp\left(-\frac{Ea}{RT}\right)$.

Where, $k_{p}$, the reaction rate constant, $C_{e}$ is the ethylene concentration, y is the catalyst concentration, $k_{po}$ is the preexponential factor (85.0 L/mol s), $E_{a}$ is 5.5 kcal/mol, ε is the prosoity [31].

### 2.2. Fluid Flow

The Navier–Stokes equations govern the movement of fluids and can be seen as an extension of Newton’s second law of motion designed explicitly for liquids. In the case of a compressible Newtonian fluid, the equations yield the following outcomes:

(4) $$\rho \left(\frac{\partial u}{\partial t}+u\cdot \nabla u\right)=-\nabla p+\nabla \cdot \left(\mu \left(\nabla u+{\left(\nabla u\right)}^{T}\right)-\frac{2}{3}\mu \left(\nabla \cdot u\right)I\right)+F$$

where, u, represents the velocity of the fluid, p represents the pressure of the fluid, ρ represents the density of the fluid, and μ represents the fluid’s dynamic viscosity. In a simplified form, assuming laminar flow and constant physical properties. $(\nabla {u)}^{T}$ is the transpose of the velocity gradient tensor. The terms on the right-hand side represent the pressure gradient force, viscous forces, and the body force term (F). The velocity profile z-direction (axial direction) can be represented as follows:

(5) $$\rho \;\left(\frac{\partial v_{z}}{\partial t}+v_{x}\frac{\partial v_{z}}{\partial x}+v_{y}\frac{\partial v_{z}}{\partial y}+v_{z}\frac{\partial v_{z}}{\partial z}\right)=-\frac{\partial p}{\partial z}+\mu \;\left(\frac{\partial^{2}v_{z}}{\partial x^{2}}+\frac{\partial^{2}v_{z}}{\partial y^{2}}+\frac{\partial^{2}v_{z}}{\partial z^{2}}\right)+\rho g_{z}$$

The initial conditions 

$v_{z}=v_{o}$ at z=0

The boundary condition.

$v_{x}=v_{y}=v_{z}=0$ at reactor walls (no-slip conditions).

### 2.3. Heat Transfer

The following expression describes the heat diffusion governing the cartesian temperature distribution for three-dimensional unsteady heat transfer involving heat generation term (q) [27].

(6) $$\rho_{i}C_{p_{i}}\frac{\partial T}{\partial t}=k_{i}\left(\frac{\partial^{2}T}{\partial x^{2}}+\frac{\partial^{2}T}{\partial y^{2}}+\frac{\partial^{2}T}{\partial z^{2}}\right)-\rho_{i}C_{p_{i}}(v_{x}\frac{\partial T}{\partial x}{+v}_{y}\frac{\partial T}{\partial y}{+v}_{z}\frac{\partial T}{\partial z})+Q_{v}$$

Initial conditions:

(7) $$T=T_{0}\;\;at\;t=0.$$

Boundary conditions

$\frac{dT}{dx}=0\;at\;x=0$ (Adiabatic walls)

Where *k* is the conductivity of the material (W·m^−1^·K^−1^), $\rho_{i}$ is the density (kg·m^−3^) of component i, ${C_{p}}_{i}$ is the specific heat capacity (J·kg^−1^·K^−1^), $Q_{v}$ is the rate at which energy is generated per unit volume of the medium (W·m^−3^). Solving the equation requires a total of six boundary conditions, two for each coordinate. The physical properties used in the simulation are listed in Table 1.

## 3. Results and Discussion

### 3.1. Temperature Distribution

**Figure 2:**
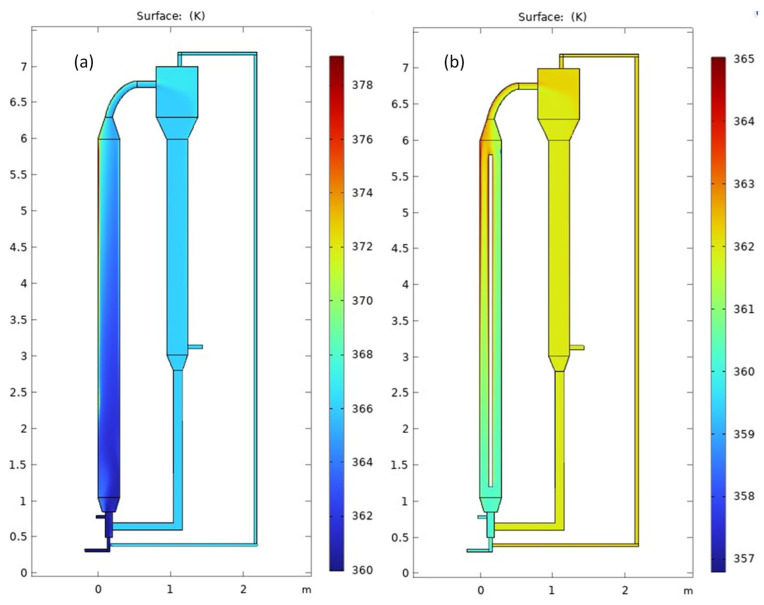
Temperature distribution profile throughout the MZCR (**a**) without a cooler, (**b**) with cooler insertion in the riser (**b**).

Figure 2 presents the surface temperature profile across the MZCR. The visual depiction in Figure 2 highlights the impact of integrating our open-loop cooler inside the riser. The diagram vividly illustrates the remarkable results achieved through the implementation of our cooling system. The figure showcases the efficient cooling effect, effectively maintaining lower temperatures with the riser. For specific model dimensions, please refer to Table 2. The visual depiction in Figure 2 allows us to witness the direct impact of our cooling system on the reactor’s performance. By effectively maintaining the temperature at the desired temperature in the riser, our open-loop cooler plays a crucial role in ensuring optimal and controlled conditions for the chemical reactions taking place in the reactor [32,33]. 

**Figure 3:**
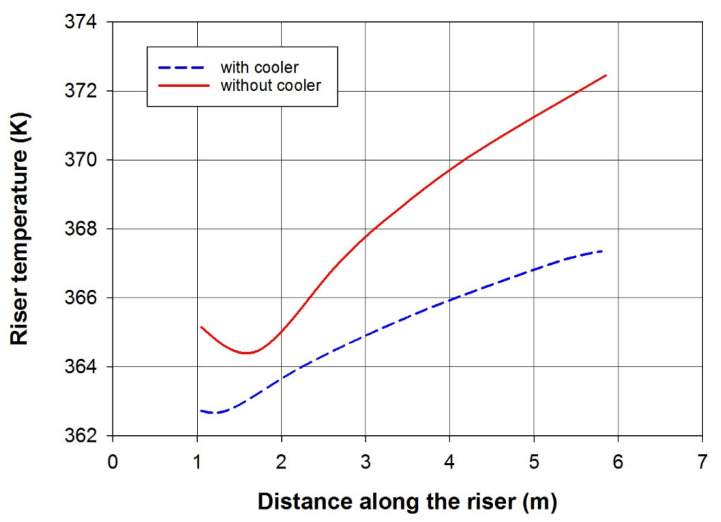
Temperature profile across the length of the riser in the presence and the absence of a cooler in the riser. Coolant and fresh feed inlet temperature is 360 K.

Figure 3 is a line graph that visually represents the impact of integrating our cooler inside the riser. It demonstrates a substantial temperature reduction of approximately 5 degrees Celsius compared to the scenario without a cooler. The diagram effectively showcases the successful results achieved with the implementation of our open-loop cooler. This cooling effect highlights the excellent performance and efficiency of our cooler in maintaining the desired temperature within the riser (around 360 K in this case). Linked to Figure 2, the gas is introduced into the lower part of the reactor at 360 K. As it moves through the rector, it combines with the recycled gas, causing its temperature to rise to approximately 365.5 K (in the scenario without a cooler). Upon entering the expanded section in the riser (at z = 1 m), the temperature initially undergoes a minor decrease before showing a steady increase, driven by the exothermic polymerization reaction. This temperature trend is also observed in the presence of a cooler. However, with the cooler, the gas temperature upon entering the riser reaches around 362.5 K, slightly higher than the inlet temperature (360 K). As the exothermic polymerization reaction commences, the temperature gradually rises to approximately 360 K, contrasting with the scenario without a cooler, where it reaches 373 K. 

### 3.2. Velocity Distribution Profile

**Figure 4:**
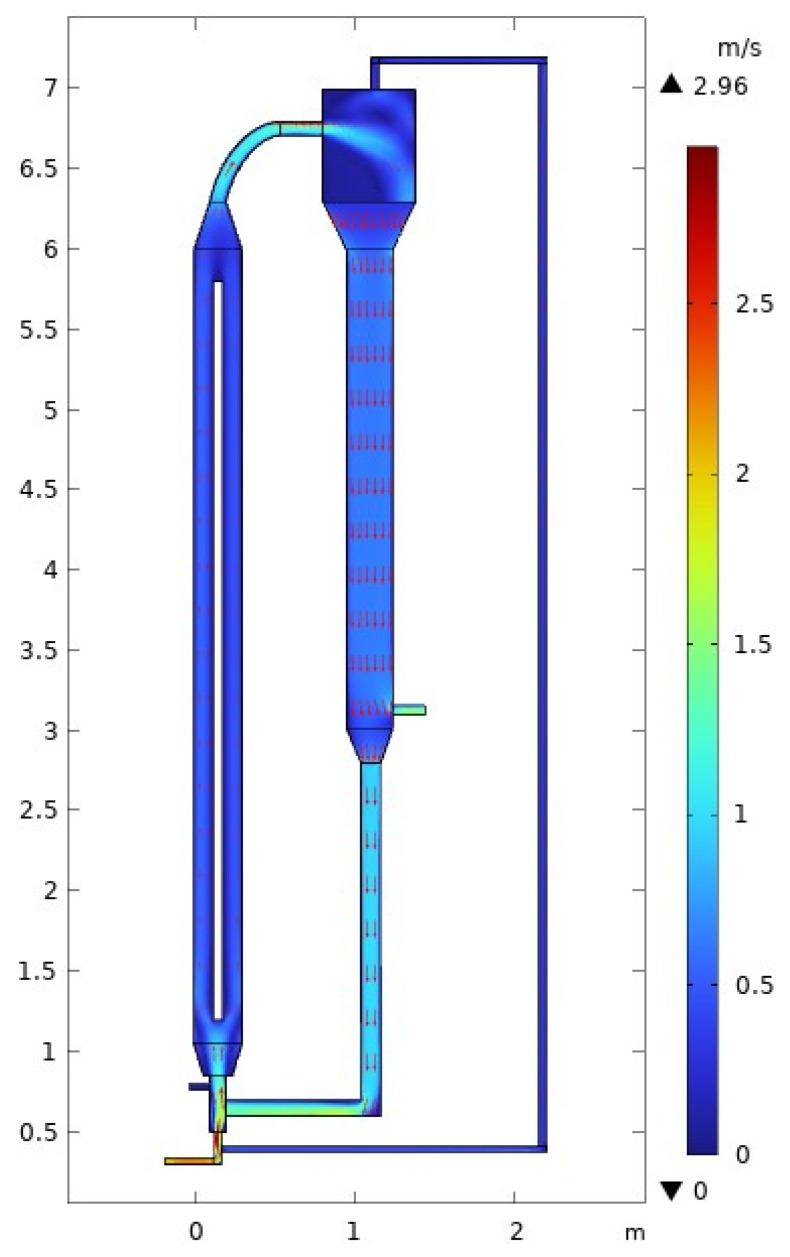
Velocity profile across the MZCR. The arrows display the velocity profile proportional to the velocity. Inlet gas velocity of 3 m/s.

Examining the surface velocity profile of Figure 4, we can analyze the velocity contour along the MZCR. To facilitate this analysis, in the CFD analysis, a cut line spanning from 1 to 5.8 m is established, beginning 1 m from the starting point. Along this cut line, we observe a distinct pattern of velocity changes. Initially, the velocity experiences a rapid increase as it progresses along the cut line. However, upon reaching the narrow area situated between the cooler and riser walls, the velocity sharply drops. This reduction can be attributed to the restricted space within this region. Moving forward, as the velocity reaches the neighborhood of the ethylene entrance, it attains its maximum value but subsequently decreases as it combines with both the gas recycle stream and the polymer stream. Along the cooler section, the velocity exhibits a slight increase due to the presence of the narrow area. Notably, a significant drop occurs after the termination of the cooler entrance (top side), where an expansion takes place, causing a further decrease in velocity. Lastly, as the velocity progresses along the elbow line, which connects the riser with the downer, there is a slight increment in velocity. This observed increase, although minimal, signifies the transition between these two components of the system. The detailed examination of the surface velocity profile along the MZCR, as depicted in Figure 4, provides valuable insights into the flow dynamics and characteristics within the system.

### 3.3. Effect of Feed Velocity

**Figure 5:**
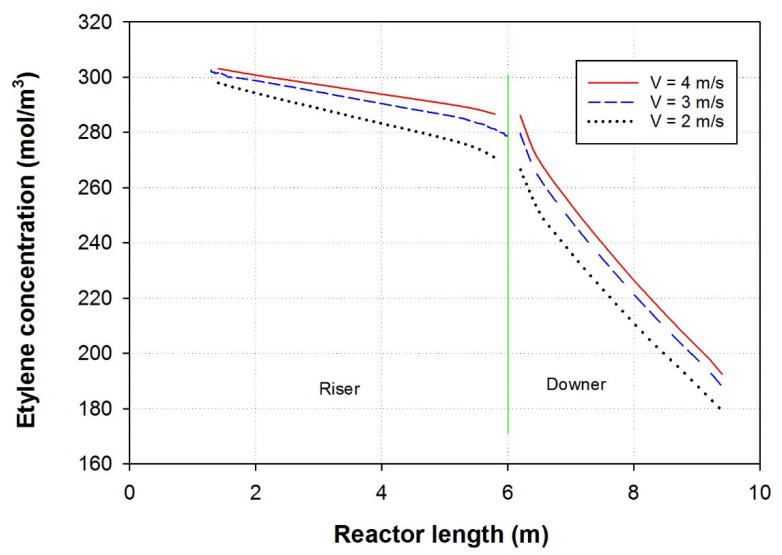
Examining the impact of riser velocity on the ethylene concentration profile along the length of the MZCR, the riser in the presence of a cooler followed by the downer.

At the inception of the process, ethylene is introduced at the lowermost part of the riser, characterized by a predetermined concentration of 0.325 mol/L. This incoming ethylene stream subsequently combines with unreacted recycled ethylene and ethylene accompanied by polymer powder. As a result, there is a marginal increase in ethylene concentration just before it enters the riser, where the actual polyethylene formation takes place throughout the reactor. Figure 5 displays the ethylene concentration profile along the river and downer. This graphical depiction provides a comprehensive view of the distribution and concentration levels of ethylene across the reactor’s length at various riser velocities. The figure presents a line graph illustrating the variations in ethylene concentration along the entire length of the riser. The combined insights from both figures offer a deeper understanding of the progression and distribution of ethylene concentration throughout the riser reactor. It allows for a comprehensive analysis of the reaction dynamics and aids in optimizing process parameters for efficient polyethylene production. As the velocity increased ethylene concentration in both riser and downer increased. That is attributed to the decrease in ethylene residence time and the consequent decrease in ethylene consumption [34,35]. With a shorter residence time, ethylene molecules have less time to react and get consumed in the polymerization process. As a result, the overall ethylene consumption in the reactor decreases. This indicates that the higher gas velocities, the polymerization reaction occurs more rapidly, leading to an increase in ethylene concentration, but at the same time, each single ethylene molecule spends less time in the reactor, resulting in reduced ethylene consumption overall [36]. 

### 3.4. Influence of Cooler Insertion

**Figure 6:**
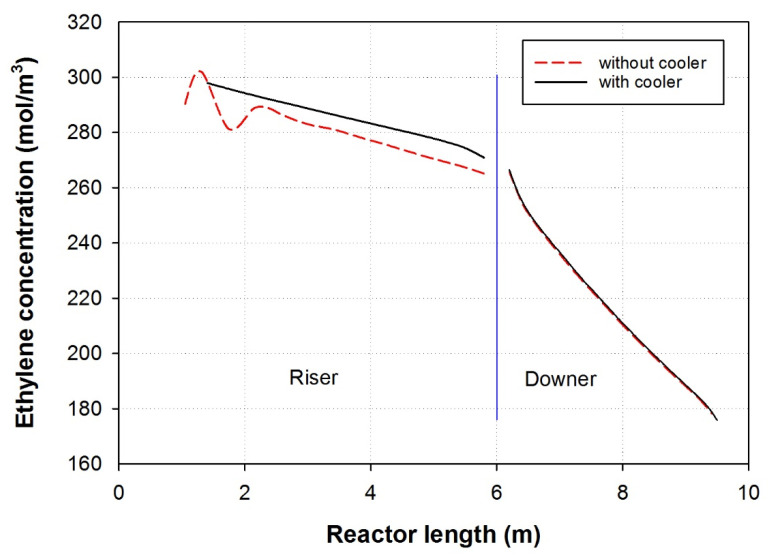
In a comparison of the ethylene concentration profile along the length of the MZCR, the riser is followed by the downer in the presence of a cooler, and without a cooler, the porosity of the Riser is 0.9. feed velocity 2 m/s.

Figure 6 illustrates a comparison of the ethylene concentration profiles along the length of the MZCR, including the riser followed by the downer, both with and without the presence of a cooler. In this study, the porosity of the riser was set to 0.9. In the case without a cooler, the ethylene concentration exhibited fluctuations at the onset of the polymerization. However, when a cooler is inserted into the system, it effectively stabilizes the reactor, eliminating these fluctuations. The scenario of a cooler inserted into the riser to regulate and control the temperature during the polymerization process, effectively stabilized the reactor’s temperature, leading to a more uniform heat distribution and reaction conditions. As a result, the ethylene concentration profile became more consistent and devoid of fluctuations. The stabilization of the ethylene concentration along the length of the reactor indicates that the polymerization process proceeded more smoothly and with better control when the cooler was employed [37,38]. The significance of these findings lies in the importance of temperature control and stability during polymerization reactions. Fluctuations in the reactant concentration can lead to inconsistent polymer properties, reduced yields, and potential operational issues in industrial settings. The insertion of a cooler in the reactor system can help ensure a more reliable and predictable reaction, leading to higher product quality and overall process efficiency [39].

### 3.5. Effect of Porosity

**Figure 7:**
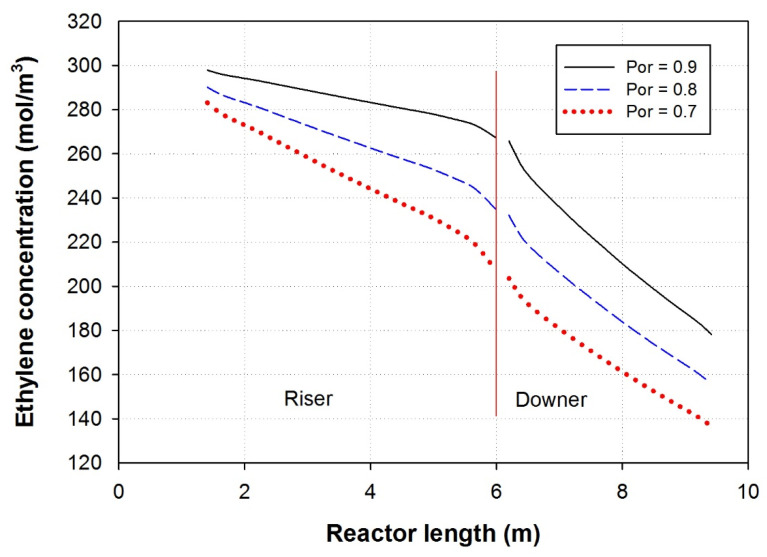
Ethylene concentration along the length of the MZCR at various riser porosity.

Porosity refers to the volume percentage of void spaces (pores) within the reactor. Porosity plays a crucial role in the mass transfer processes within the riser and the fluid dynamics. The Riser typically operates as a fluidized bed reactor, and porosity plays a significant role in the mass transfer phenomena and the fluid dynamics. A decrease in the porosity in the riser increases the rate of ethylene consumption [9]. The lower porosity leads to a more uniform residence time distribution of reactants within the riser. Hence, all the reactants, including ethylene, spend a similar amount of time in the reactor, leading to a more consistent and efficient conversion process. With decreased porosity, the reactant is likely to come into direct contact with catalyst particles more frequently, increasing the likelihood of successful reaction and ethylene single-pass conversion [40,41]. When the porosity of the rector is reduced, there are fewer empty spaces for the reactants to flow through, leading to uniform residence time distribution [42]. This results in a more even distribution of the reactant within the reactor’s riser, which is the part of the reactor where the reactions take place (Figure 7). As a result, all the reactants, including ethylene, spend a similar amount of time in the reactor before exiting, leading to a more consistent residence time distribution. This is important because it ensures that each molecule of the reactant is exposed to the same reaction conditions, which promotes a more predictable and controlled reaction process [43]. Uniform residence time distribution is essential for scaling up the reaction process from lab-scale to industrial production. In large-scale reactors, maintaining a consistent residence time for all reactant molecules becomes more challenging due to increased turbulence and missing. By understanding the impact of gas velocity on residence time, engineers can optimize and design industrial reactors to achieve the desired residence time distribution, ensuring a smooth and efficient reaction at large scales [44].

**Figure 8:**
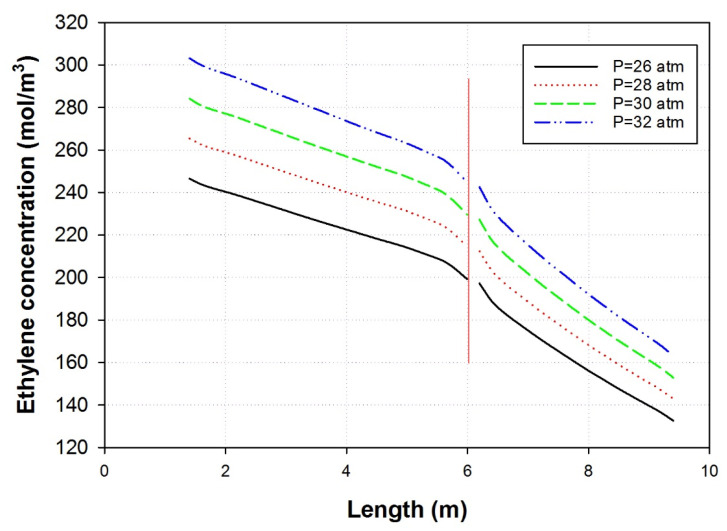
Effect of reactor pressure on ethylene concentrations inside the riser and downer zones of the MZCR.

Figure 8 shows the effect of pressure on the riser and the downer of the MZCR. The figure shows that the increase in pressure increases the ethylene concentration in the reactor and hence helps in increasing the reaction rate and the production of polyethylene. In general, Figure 8 illustrates how higher pressure can positively affect the performance of the MZCR. By adjusting the pressure, we can optimize the reactor to produce polyethylene and improve efficiency. However, it is worth noting that excessive pressure increases may not always yield benefits and could introduce challenges that require careful consideration during reactor design and operation.

## 4. Conclusions

The Multizone Circulating Reactor (MZCR) shows promise in converting ethylene to polyethylene by incorporating zones like the riser and downer that allow for effective interactions between the reactants and catalysts. Adding a cooler to the system offers stability advantages while maintaining conversion efficiency in the riser. Moreover, the porosity within the riser plays a role in influencing ethylene conversion rates. Optimizing this porosity improves diffusion, mass transfer and catalyst utilization ultimately leading to ethylene conversion in the MZCR. Looking ahead there are opportunities for optimizing and advancing the MZCR process. Researchers and engineers can explore cooling techniques and design modifications to enhance system stability and productivity. By tuning the porosity of the riser, we can achieve higher ethylene conversion rates allowing us to tailor reactor performance based on specific production requirements. Future research should focus on characterizing how catalyst particle size, operating conditions, and specific reaction kinetics interact within the MZCR. Taking this approach will help develop guidelines for selecting optimal porosity and operating parameters that result in the best ethylene conversion rates. In general, the MZCR shows potential in the polyethylene manufacturing industry. It provides a platform for progress and sustainable enhancements in process efficiency. By tackling obstacles and optimizing factors the MZCR has the potential to be a competitive and eco-friendly technology for producing polyethylene in the coming years.

## Figures and Tables

**Table 1:** Physical properties of polymers and monomers [22].

Parameter	Value
Pressure (atm)	30
Gas density (kg/m^3^)	29
Gas viscosity (μPa.s)	11.6
Polymer density (kg/m^3^)	950
Polymer heat capacity (J/g K)	1.839
Ethylene heat capacity (J/mol K)	46
Nitrogen heat capacity (J/mol K)	28
Gas thermal conductivity (W/mol K)	0.03015
Polymer thermal conductivity (W/mol K)	0.34
The heat of reaction (kJ/mol)	94.5

**Table 2:** Design specifications of the MZCR for polyethylene production [9].

Parameter	Values
Riser length (m)	5
Downer length (m)	3
Riser diameter (m)	0.3
Downer diameter (m)	0.3
Riser void fraction	0.9
Downer void fraction	0.15

## Data Availability

This study does not involve the use of any specific data as it is based solely on simulation.

## References

[1] Guarino V., Marrese M., Ambrosio L. (2014). Chemical and Physical Properties of Polymers for Biomedical Use.

[2] Yang Y.-N., Liu H., Zhu L.-T., Jin J., Zhang X.-B., Luo Z.-H. (2023). Simulation of Molecular and Particle Properties of Polypropylene Produced in Multizone Circulating Reactors. Ind. Eng. Chem. Res..

[3] Hutley T.J., Ouederni M., Al-Ali AlMa’adeed M., Krupa I. (2016). Polyolefins—The History and Economic Impact BT—Polyolefin Compounds and Materials: Fundamentals and Industrial Applications.

[4] Ghasem N. (2020). CO_2_ removal from natural gas. Advances in Carbon Capture.

[5] Ghasem N. (2019). Modeling and Simulation of CO_2_ Absorption Enhancement in Hollow-Fiber Membrane Contactors using CNT–Water-Based Nanofluids. J. Membr. Sci. Res..

[6] Sui Y., Qiu Z., Liu Y., Li J., Cui Y., Wei P., Cong C., Meng X., Zhou Q. (2023). Ultra-high molecular weight polyethylene (UHMWPE)/high-density polyethylene (HDPE) blends with outstanding mechanical properties, wear resistance, and processability. J. Polym. Res..

[7] Covezzi M., Mei G. (2001). The multizone circulating reactor technology. Chem. Eng. Sci..

[8] Fernandas F.A.N., Lona L.M.F. (2004). Multizone circulating reactor modeling for gas-phase polymerization. II. Reactor operating with gas barrier in the downer section. J. Appl. Polym. Sci..

[9] Fernandas F.A.N., Lona L.M.F. (2004). Multizone circulating reactor modeling for gas-phase polymerization. I. Reactor modeling. J. Appl. Polym. Sci..

[10] Ghasem N.M. (1999). Effect of polymer particle size and inlet gas temperature on industrial fluidized bed polyethylene reactors. Chem. Eng. Technol..

[11] Ghasem N.M. (2006). Design of a fuzzy logic controller for regulating the temperature in industrial polyethylene fluidized bed reactor. Chem. Eng. Res. Des..

[12] Whitfield R., Truong N.P., Messmer D., Parkatzidis K., Rolland M., Anastasaki A. (2019). Tailoring polymer dispersity and shape of molecular weight distributions: Methods and applications. Chem. Sci..

[13] Kulajanpeng K., Sheibat-Othman N., Tanthapanichakoon W., McKenna T.F.L. (2022). Multiscale modelling of multizone gas phase propylene (co)polymerization reactors—A comprehensive review. Can. J. Chem. Eng..

[14] Ge S., Lou Z., Yang Y., Huang Z., Sun J., Wang J., Yang Y. (2020). Electrostatic effects on hydrodynamics in the riser of the circulating fluidized bed for polypropylene. AIChE J..

[15] Che Y., Tian Z., Liu Z., Zhang R., Gao Y., Zou E., Wang S., Liu B. (2015). A CFD-PBM Model Considering Ethylene Polymerization for the Flow Behaviors and Particle Size Distribution of Polyethylene in a Pilot-Plant Fluidized Bed Reactor. Powder Technol..

[16] Yan W.-C., Li J., Luo Z.-H. (2012). A CFD-PBM coupled model with polymerization kinetics for multizone circulating polymerization reactors. Powder Technol..

[17] Yan W.-C., Chen G.-Q., Luo Z.-H. (2012). A CFD modeling approach to design a new gas barrier in a multizone circulating polymerization reactor. Ind. Eng. Chem. Res..

[18] Upadhyay M., Kim A., Kim H., Lim D., Lim H. (2020). An Assessment of Drag Models in Eulerian–Eulerian CFD Simulation of Gas–Solid Flow Hydrodynamics in Circulating Fluidized Bed Riser. ChemEngineering.

[19] Ngo S.I., Lim Y.-I. (2020). Multiscale Eulerian CFD of Chemical Processes: A Review. ChemEngineering.

[20] Emami M., Parvazinia M., Abedini H. (2017). Gas-phase polymerization of propylene at low reaction rates: A precise look at catalyst fragmentation. Iran. Polym. J..

[21] Wu G.-L., Tian Z., Wang J.-J., Gu X.-P., Feng L.-F. (2011). Simulation of multizone circulating reactor for propylene gas-phase polymerization. Huaxue Gongcheng/Chem. Eng..

[22] Adli H., Mostoufi N., Ghafelebashi S.M. (2011). Modeling of a multizone gas-phase polyethylene reactor with a cluster-based approach. J. Appl. Polym. Sci..

[23] Wei L.-H., Yan W.-C., Luo Z.-H. (2011). A preliminary CFD study of the gas-solid flow fields in multizone circulating polymerization reactors. Powder Technol..

[24] Mei G., Beccarini E., Caputo T., Fritze C., Massari P., Agnoletto D., Pitteri S. (2009). Recent technical advances in polypropylene. J. Plast. Film Sheeting.

[25] Ghasem N.M., Ang W.L., Hussain M.A. (2009). Dynamics and stability of ethylene polymerization in multizone circulating reactors. Korean J. Chem. Eng..

[26] Ghasem N.M., Ang W.-L., Hussain M.A. (2008). Dynamic model for polyethylene production in a multizone circulating reactor. Chem. Prod. Process Model..

[27] Santos J.L., Asua J.M., De La Cal J.C. (2006). Modeling of olefin gas-phase polymerization in a multizone circulating reactor. Ind. Eng. Chem. Res..

[28] Wu C., Chen W. (2006). Recent advances in technology of impact PP prepared in-situ in reactor. Hecheng Shuzhi Ji Suliao/China Synth. Resin Plast..

[29] Marandi R., Kamyabi M., Mostoufi N. (2018). Hydrodynamic design of multi-zone circulating reactors using CFD. Can. J. Chem. Eng..

[30] Dompazis G., Kanellopoulos V., Touloupides V., Kiparissides C. (2008). Development of a multi-scale, multi-phase, multi-zone dynamic model for the prediction of particle segregation in catalytic olefin polymerization FBRs. Chem. Eng. Sci..

[31] Dadebo S.A., Bell M.L., McLellan P.J., McAuley K.B. (1997). Temperature control of industrial gas phase polyethylene reactors. J. Process Control.

[32] Xiong C., Bai L., Li H., Guo Y., Yu Y., Lin G. (2022). Experimental study on a R134a loop heat pipe with high heat transfer capacity. Heat Mass Transf..

[33] Wu D., Wu W. (2019). Battery Powered Portable Thermal Cycler for Continuous-Flow Polymerase Chain Reaction Diagnosis by Single Thermostatic Thermoelectric Cooler and Open-Loop Controller. Sensors.

[34] Liu Y., Wu Y., Shi X., Wang C., Gao J., Lan X. (2020). 3D CPFD Simulation of Circulating Fluidized Bed Downer and Riser: Comparisons of Flow Structure and Solids Back-Mixing Behavior. Processes.

[35] Chalermsinsuwan B., Gidaspow D., Piumsomboon P. (2013). Comparisons of particle cluster diameter and concentration in circulating fluidized bed riser and downer using computational fluid dynamics simulation. Korean J. Chem. Eng..

[36] Kissin Y.V., Mink R.I., Nowlin T.E., Brandolini A.J., Kaminsky W. (1999). Kinetics and Mechanism of Ethylene Polymerization and Copolymerization Reactions with Heterogeneous Titanium-Based Ziegler-Natta Catalysts BT—Metalorganic Catalysts for Synthesis and Polymerization.

[37] Thakur A.K., Gupta S.K., Chaudhari P. (2020). Modeling and simulation of an industrial slurry phase ethylene polymerization reactor: Effect of reactor operating variables. Iran. Polym. J..

[38] Deng B., Jiang Y., Gao L., Zhao B. (2023). CFD modeling of ethylene degradation in gas-phase photocatalytic reactors. Environ. Sci. Pollut. Res..

[39] Li C., Ni L., Chen Q., Jiang J., Zhou K. (2022). Temperature Control of Exothermic Reactions Using n-Octadecane@MF Resin microPCMs Based on Esterification Reactions. Processes.

[40] Zhang Z., Jiang B., He F., Fu Z., Xu J., Fan Z. (2019). Comparative Study on Kinetics of Ethylene and Propylene Polymerizations with Supported Ziegler–Natta Catalyst: Catalyst Fragmentation Promoted by Polymer Crystalline Lamellae. Polymers.

[41] Ahsan Bashir M., McKenna T.F.L., Pauer W. (2018). Reaction Engineering of Polyolefins: The Role of Catalyst Supports in Ethylene Polymerization on Metallocene Catalysts BT—Polymer Reaction Engineering of Dispersed Systems: Volume I.

[42] Sans V., Karbass N., Burguete M.I., García-Verdugo E., Luis S. (2012). V Residence time distribution, a simple tool to understand the behaviour of polymeric mini-flow reactors. RSC Adv..

[43] Lovreglio P., Buist K.A., Kuipers J.A.M., Peters E.A.J.F. (2022). Analysis of Particle-Resolved CFD Results for Dispersion in Packed Beds. Fluids.

[44] Johnson M.D., May S.A., Groh J.M.C., Webster L.P., Shankarraman V., Spencer R.D., Luciani C.V., Polster C.S., Braden T., Nagy Z.K., El Hagrasy A., Litster J. (2020). Understanding Residence Time, Residence Time Distribution, and Impact of Surge Vessels BT—Continuous Pharmaceutical Processing.

